# Long-Term Kidney Prognosis and Pathological Characteristics of Late-Onset Lupus Nephritis

**DOI:** 10.3389/fmed.2022.882692

**Published:** 2022-05-30

**Authors:** Na Tian, Qian Zhou, PeiRan Yin, WenFang Chen, LingYao Hong, QiMei Luo, MengHua Chen, XueQing Yu, Wei Chen

**Affiliations:** ^1^Department of Nephrology, The First Affiliated Hospital, Sun Yat-sen University, Key Laboratory of National Health Commission, Guangdong Provincial Key Laboratory of Nephrology, Guangzhou, China; ^2^Department of Nephrology, General Hospital of Ningxia Medical University, Ningxia, China; ^3^Department of Medical Statistics, Clinical Trials Unit, The First Affiliated Hospital of Sun Yat-sen University, Guangzhou, China; ^4^Department of Nephrology, Second Affiliated Hospital of Soochow University, Suzhou, China; ^5^Department of Pathology, The First Affiliated Hospital, Sun Yat-sen University, Guangzhou, China; ^6^Guangdong Provincial People's Hospital, Guangzhou, China

**Keywords:** lupus nephritis, late-onset, kidney outcome, renal pathology, Chinese population

## Abstract

**Background:**

Arguments still exist on prognosis of late-onset SLE, especially their kidney function. The purpose of this study was to investigate long-term kidney outcomes in patients with late-onset lupus nephritis (LN).

**Methods:**

A retrospective long-term cohort study was conducted in adult Chinese patients with LN. The patients were divided into late- (>50 years) and early-onset (<50 years) LN groups. The baseline characteristics, especially the kidney pathological characteristics, were compared. The cohort was followed-up for kidney outcome defined as doubling of serum creatinine or ESRD. Cox regression analysis was used to examine the association between late onset LN and its outcomes.

**Results:**

A total of 1,264 patients were recruited, who were assigned to late-onset LN with 102 patients and early-onset LN with 1,162 patients. The late-onset LN group showed a worse baseline kidney function and more chronic pathological lesions than the early-onset LN group. During a follow-up time of 55 (3, 207) months, 114 (13.1%) deaths occurred, 107 (12.2%) had doubling of creatinine, and 80 (9.1%) developed end-stage kidney disease. The 5- and 10-year survival rates of the late-onset LN group were 67.6 and 50.5%, respectively, which were much worse than those of the early-onset LN group (89.8 and 84.6%, respectively). However, no significant difference was found on kidney survival (log-rank chi-square = 3.55, *p* = 0.06). Cox regression analysis showed that late-onset LN was an independent risk factor for patient survival (hazard ratio = 3.03, 95% CI (1.39, 6.58), *p* = 0.005). Increased baseline serum creatinine was an independent risk factor for kidney survival of patients with late-onset LN.

**Conclusions:**

Patients with late-onset LN had milder active lesions but severer chronic lesions in kidney pathology. They have poorer overall outcome but relatively favorable kidney outcome.

**Trial Registration:**

ClinicalTrials.gov Identifier: NCT03001973, 22 December 2016 retrospectively registered.

## Introduction

Systemic lupus erythematosus (SLE) is the most common cause (54.3%) of secondary glomerular nephropathy in China (1). SLE mainly occurs in young women of child-bearing age and is not commonly found in elderly population. Previous studies suggested that the onset age of lupus is an important risk factor associated with clinical manifestations and outcomes (2–6). Two nested case-control study from Brazil and US, respectively, Appenzeller et al. (7), Bertoli et al. (8) found that patients with late-onset SLE have milder symptoms, but present higher rate of organ damage and mortality than patients with early-onset SLE. While another found that late-onset SLE has a benign prognosis (9). In a Chinese study, kidney pathology analysis showed that activity lesions are milder and chronic lesions are more severe in patients with late-onset SLE compared with those in patients with early-onset SLE, but no difference in kidney outcomes was found between the two groups (3).

Although certain differences between late- and early-onset SLE have been reported, few distinct patterns of clinical manifestation, therapeutic response, or prognosis have been confirmed. In addition, to the best of our knowledge, only a few studies have investigated kidney pathological features and kidney outcome in population with late-onset lupus nephritis (LN). Therefore, we aimed to assess the kidney pathological characteristics and outcomes of patients with late-onset LN based on a large sample size and long-term follow-up data.

## Materials and Methods

### Subjects

A single center, retrospective cohort study was designed. Clinical and kidney histopathological data were extracted from the LN database (ln.medidata.cn) of the Department of Nephrology, the First Affiliated Hospital of Sun Yat-sen University. Patients diagnosed with LN at the age of 16 years or older from January 1, 1996 to December 31, 2011 were enrolled. All patients were diagnosed using the 1997 revised SLE criteria of the American College of Rheumatology (10). Patients who had end-stage renal disease (ESRD), cancer, or drug-induced LN at the time of diagnosis were excluded. Each biopsy specimen with at least 10 glomeruli was included for histopathological analysis. The protocol was approved by the human ethics committee of the First Affiliated Hospital, Sun Yat-sen University. Written informed consent was obtained from each participant.

The cut-off age of 50 was employed referring to the previous studies to define the late-onset LN (11–15). Patients with LN aged 50 years or older at kidney biopsy were assigned to the late-onset LN group, whereas those with LN aged <50 years at kidney biopsy were assigned to the early-onset LN group (control).

### Data Collection and Clinical Definitions

Age at onset was obtained in a retrospective manner as the first sign of kidney involvement was detected, including proteinuria ≥0.5 g/24 h, glomerular hematuria, and cellular cast. Cardiovascular complications included coronary heart disease, chronic heart failure, arrhythmia, and cardiomyopathy. Organ damage was assessed using the Systemic Lupus Erythematosus International Collaborating Clinics/American College of Rheumatology Damage Index (SDI) (16). To be scored, each manifestation in the SDI is required to be present for at least 6 months unless it is noted in the SDI instructions. Disease activity was evaluated using the SLE disease activity index (SLEDAI) (17). Acute kidney injury (AKI) was diagnosed based on 2012 KDIGO criteria (18). Estimated glomerular filtration rate (eGFR) was calculated using the Chronic Kidney Disease Epidemiology Collaboration equation (19).

Therapeutic variables included exposure to glucocorticoid at induction treatment, dose of prednisone at induction, high-dose glucocorticoid at first diagnosed (pulse therapy or oral prednisone ≥1 mg/kg day), immunosuppressive agents treatment (taken methotrexate, mycophenolate mofetil, cyclophosphamide, azathioprine, or combination), and use of angiotensin converting enzyme inhibitor (ACEI)/angiotensin receptor blocker (ARB).

Kidney histopathological data were reviewed by an experienced pathologist according to the 2003 International Society of Nephrology/Kidney Pathology Society classification of LN (20).

### Study Outcomes

All the participants were followed up for at least 1 year until December 31, 2013. Patients were required to return to our hospital at least once a year for an overall medical evaluation and/or were interviewed annually through phone call by experienced doctors to assess the general conditions. The primary endpoint was a composite of kidney outcomes, including doubling of serum creatinine (serum creatinine doubled compared with the baseline) and ESRD (eGFR ≦15 mL/min; persistent dialysis; kidney transplant). The secondary endpoint was all cause mortality.

### Statistical Analysis

Patient characteristics were presented as mean ± SD for normally distributed continuous variables, median (interquartile range) for skewed continuous variables, and frequencies and percentages for categorical variables. Comparisons between the late- and control groups were performed using the Student's t-test for normally distributed continuous variables, the Mann–Whitney U-test for non-normally distributed continuous variables, and the Chi-square test for categorical variables. Patients' cumulative survival and kidney survival rates (at a combined kidney endpoint of creatinine doubling and ESRD) were calculated using Kaplan–Meier curves. Unadjusted and adjusted Cox proportional hazards regression models were used to evaluate the risk factors for mortality in all participants and kidney mortality in patients with late-onset LN. Potential confounders were included in univariate Cox regression analysis. Significant variates with a *p*-value <0.10 in the univariate analysis were forced into the multivariate models. Other variates were selected into the multivariable models using the forward method (entry: 0.1, removal: 0.2). To adjust the impact of age on patient survival, we collected the data of life expectancy at LN onset for each participant based on the World Bank latest report (The World Bank. Data of life expectancy at birth, total (years), China (2019). https://data.worldbank.org/indicator/SP.DYN.LE00.IN?end=2016&locations=CN&start=1960&view=chart&year_low_desc=true). Since only data from 1960 to 2016 could be obtained, we estimated life expectancy with the recent 5-year life growth rate of 0.21% for those who were born between 1920 and 1959. In the multivariable Cox regression, life expectancy was considered as a confounder into model to adjust the impact of age to patient survival.

A two-sided *p*-value <0.05 was considered statistically significant. Statistical analysis was performed using SPSS software, version 20.0 (SPSS, Inc., Chicago, IL, USA).

## Results

### Clinical Characteristics of Late-Onset LN

**Figure 1:**
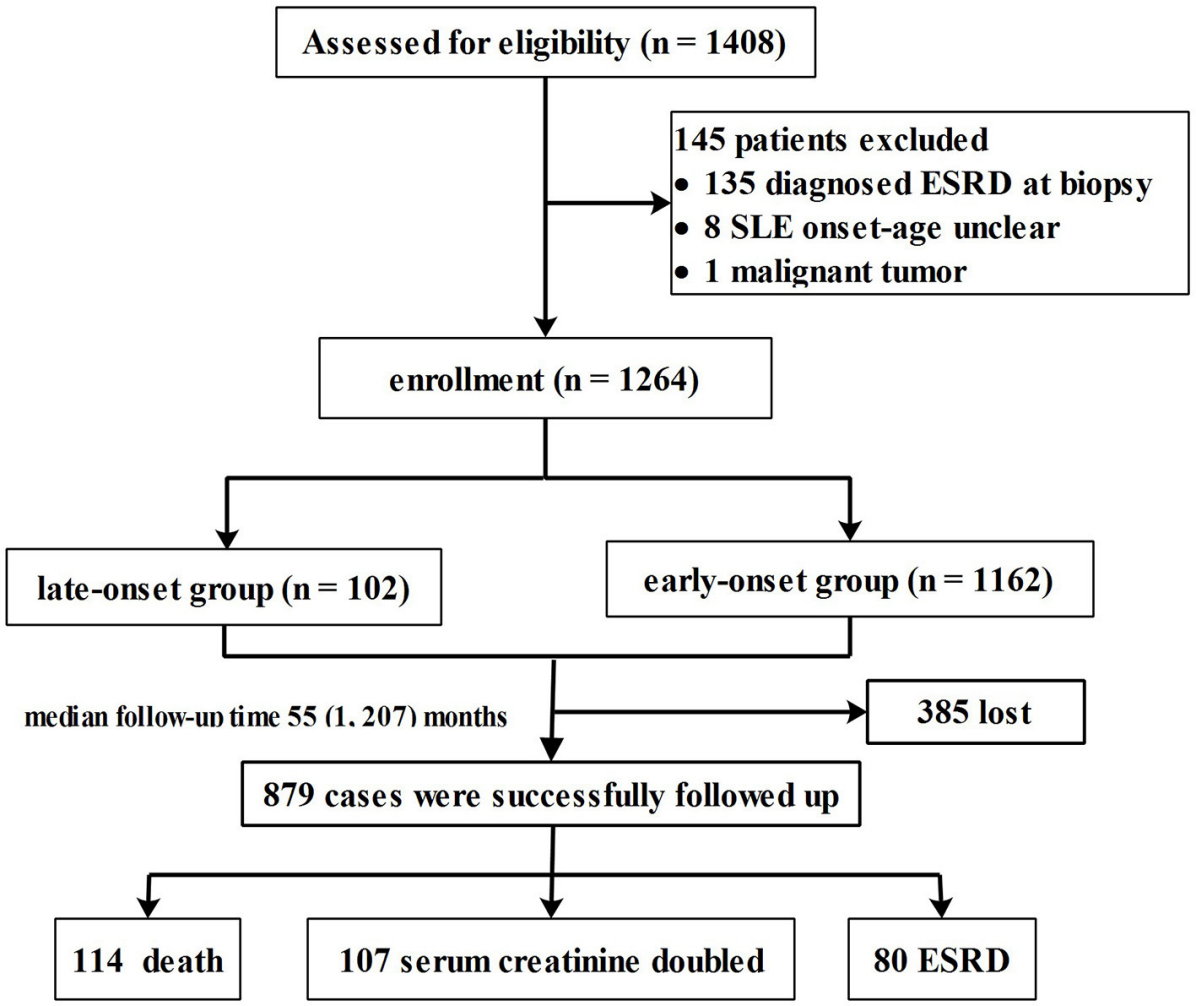
Enrollment and follow-up of study population.

A total of 1,264 patients who met the inclusion criteria were enrolled from the 1,408 patients diagnosed with LN during the study period (Figure 1). Among the eligible patients, 102 (8.1%) were assigned to the late-onset LN group and 1,162 (91.9%) to the early-onset LN group.

Tables 1, 2 showed the baseline of the two groups. The male-to-female ratio in the late-onset LN group was 2:5, which was twice of that in the early-onset LN group (*p* = 0.001). More patients with late-onset LN had hypertension and cardiovascular disease (CVD) complications compared with the patients in the early-onset LN group (*p* < 0.001). Diabetes percentage was similar in both groups. Higher percentage of patients with late-onset LN had no skin or mucous lesions. The serositis frequency was lower in the late-onset LN group. The SDI and SLEDAI scores indicated no difference between the two groups. The baseline kidney function of the late-onset LN group was significantly worse, presenting more urine protein (*p* = 0.03), smaller kidney size, and lower eGFR (*p* < 0.001). AKI occurred more frequently in the late-onset LN group (*p* = 0.005).

**Table 1:** Comparisons of baseline clinical manifestations between late- and early-onset LN groups.

**Items**	**All**	**Early-onset LN group**	**Late-onset LN group**	***p***
N	1,264	1,162	102	
Male/female	1:5	1:5	2:5	0.001
Age at diagnosis (years)	31.12 ± 12.21	28.81 ± 9.99	58.13 ± 6.80	<0.001
Duration before diagnosis (months)	4 (1, 24)	3 (1, 20)	3 (1, 12)	0.37
Hypertension, *n* (%)	35 (2.7)	21 (1.8)	14 (13.7)	<0.001
CVD history, *n* (%)	82 (6.5)	61 (5.2)	21 (20.6)	<0.001
Diabetes history, *n* (%)	18 (1.4)	13 (1.1)	5 (4.9)	0.12
BMI (kg/m^2^)	21.09 ± 3.07	21.03 ± 3.12	21.85 ± 2.31	0.15
Fever, *n* (%)	415 (32.8)	389 (33.4)	26 (25.4)	0.11
Edema, *n* (%)	834 (65.9)	771 (66.3)	63 (61.7)	0.45
**Skin and mucous**, ***n*** **(%)**
None	161 (13.2)	123 (10.6)	38 (37.2)	0.01
Rash	568 (44.7)	527 (45.3)	41 (40.2)	0.34
Alopecia	249 (19.6)	238 (20.5)	11 (10.8)	0.01
Mucous ulcer	121 (9.5)	115 (9.9)	6 (5.9)	0.19
Raynaud's phenomenon	18 (1.4)	18 (1.6)	0 (0)	0.20
Photosensitivity	147 (11.6)	141 (12.1)	6 (5.9)	0.06
**Musculoskeletal**, ***n*** **(%)**
None	769 (60.8)	717 (61.7)	52 (51.0)	0.07
Arthritis	431 (34.1)	391 (33.6)	40 (39.2)	0.23
Myalgia	40 (3.2)	34 (2.9)	6 (5.9)	0.09
Muscle weakness	24 (1.9)	20 (1.7)	4 (3.9)	0.11
Serositis, *n* (%)	755 (59.8)	751 (64.7)	53 (51.2)	0.04
Systolic BP (mmHg)	127.7 ± 21.80	126.53 ± 21.40	138.48 ± 20.50	<0.001
Diastolic BP (mmHg)	81.45 ± 14.70	81.45 ± 14.76	79.81 ± 13.50	0.28
Hepatomegaly, *n* (%)	11 (0.8)	10 (0.9)	1 (0.9)	0.90
Splenomegaly, *n* (%)	7 (0.5)	7 (0.6)	0	0.43
SDI score	2.1 ± 1.9	2.0 ± 1.8	2.3 ± 1.9	0.12
SLEDAI score	14.74 ± 5.35	14.77 ± 5.33	14.35 ± 5.69	0.59

*CVD, cardiovascular disease; BP, blood pressure; SDI, systemic damage index; SLEDAI, systemic lupus erythematosus disease activity index*.

**Table 2:** Comparisons of baseline examination profiles between late- and early-onset LN groups.

**Items**	**All**	**Early-onset LN group**	**Late-onset LN group**	***p***
Hemoglobin (g/L)	97.81 ± 24.01	105.79 ± 24.87	96.98 ± 25.79	0.002
Serum creatinine (μmol/L)	87.0 (63.0,147.0)	85.0 (62.0,138.0)	112.0 (79.0,242.2)	<0.001
Serum albumin (g/L)	27.53 ± 7.44	27.48 ± 7.49	28.28 ± 6.98	0.29
hs-CRP (g/L)	2.52 (0.84, 7.15)	2.44 (0.84, 2.44)	3.05 (1.21, 10.90)	0.63
ESR (mm/h)	38.0 (19.0,63.0)	38.0 (20.0,62.0)	40.5 (16.0,73.0)	0.49
Anti-ds DNA positive, *n* (%)	941 (76.6)	863 (76.5)	78 (77.2)	0.87
Anti-SSA positive, *n* (%)	536 (44.7)	481 (43.7)	55 (56.7)	0.02
Anti-SSB positive, *n* (%)	241 (20.2)	217 (19.9)	24 (24.2)	0.29
Low C3, *n* (%)	994 (81.5)	915 (81.6)	79 (79.8)	0.65
Urine protein (g/24 h)	1.63 (0.72, 3.34)	1.70 (0.74, 3.41)	1.15 (0.67, 2.46)	0.02
**Left kidney size (mm)**
Length	108.48 ± 11.82	108.85 ± 11.67	101.38 ± 22.22	<0.001
Width	50.38 ± 7.06	50.52 ± 7.21	47.47 ± 10.66	<0.001
**Right kidney size (mm)**
Length	105.44 ± 16.14	105.67 ± 16.33	101.67 ± 13.87	0.02
Width	47.12 ± 7.43	49.32 ± 8.01	46.81 ± 7.34	0.01
AKI, *n* (%)	197 (16.3)	173 (15.7)	24 (23.5)	0.04
eGFR (ml/min/1.73 m^2^)	100.8 (54.37, 128.03)	104.9 (58.28, 129.24)	59.59 (25.17, 93.81)	<0.001

*ESR, erythrocyte sedimentation rate; hs-CRP, high-sensitivity C-reactive protein; ANA, anti-nuclear antibody; AKI, acute kidney injury; eGFR, estimated glomerular filtration rate*.

As for the treatment, patients with late-onset LN were administered lower dose of prednisone at the induction phase. Immunosuppressant drugs and angiotensin-converting enzyme inhibitor/angiotensin receptor blocker drugs were used in less proportion of patients with late-onset LN (Table 3).

**Table 3:** Comparisons of treatment regimen between late- and early-onset LN groups.

**Presentations**	**All**	**Early-onset LN group**	**Late-onset LN group**	***p***
*N*	1,264	1,162	102	
Glucocorticoid at induction treatment, *n* (%)	1,220 (98.3)	1,126 (98.5)	94 (95.9)	0.06
Dose of prednisone at induction (mg/d)	50.4 ± 5.6	54.1 ± 5.8	40.1 ± 4.7	<0.001
High-dose glucocorticoid at first diagnosed, *n* (%)	835(66.1)	784(67.5)	51(49.6)	<0.001
Immunosuppression drug at
induction treatment, *n* (%)	671 (53.2)	629 (54.3)	42 (41.2)	0.01
ACEI/ARB, *n* (%)	669 (52.9)	607 (52.2)	62 (60.7)	0.04

*ACEI, angiotensin converting enzyme inhibitor; ARB, angiotensin receptor blocker*.

### Kidney Histopathological Evaluation

Kidney pathological analysis was conducted in 784 eligible patients. The proportion of biopsied patients showed no statistical difference between the late- and early-onset LN groups (*p* = 0.07). The distribution of LN pathological type (ISN/RPS, 2003) was similarly between the two groups. It generally showed that chronic lesions (glomerular sclerosis, interstitial fibrosis, tubular atrophy, and arterial wall thickening) were more severe in the late-onset LN group (all *p*-value <0.05). Active lesions (including crescents, karyorrhexis, capillary tuft necrosis, glomerular capsule adhesion, subendothelial hyaline deposits and tubular necrosis) were not statistically different between the two groups (all *p*-value > 0.05) (Table 4).

**Table 4:** Kidney pathological characteristics of late-onset LN.

**Items**	**All**	**Early-onset group**	**Late-onset group**	***p***
Number of biopsy, *n* (%)	784 (62.0)	726 (62.4)	58 (56.8)	0.07
LN classification, *n* (%)				
Class II	71 (9.6)	68 (10)	3 (5.5)	0.274
Class III	81 (11.0)	73 (10.7)	8 (14.5)	0.383
Class IV	308 (41.8)	287 (42.1)	21 (38.2)	0.567
Class V	105 (14.3)	96 (14.1)	9 (16.4)	0.644
Class V+III	58 (7.9)	52 (7.6)	6 (10.9)	0.386
Class V+IV	98 (13.3)	93 (13.7)	5 (9.1)	0.338
Class VI	9 (1.3)	8 (1.2)	1 (1.8)	0.676
Glomerular sclerosis (%)	0 (0,9)	0 (0,7.4)	10.7 (0,23.7)	<0.001
Crescents (%)	3 (0,16)	3 (0,16)	0 (0,23)	0.72
Endocapillary hypercellularity, *n* (%)				0.058
None	207 (26.4)	194 (26.7)	13 (22.4)	
(25–50%)	360 (45.9)	325 (45.9)	35 (60.3)	
(>50%)	217 (27.7)	207 (28.5)	10 (17.2)	
Glomerular leukocyte infiltration, *n* (%)				0.037
None	286 (36.5)	263 (36.2)	23 (39.7)	
(<25%)	314 (40.1)	284 (39.1)	30 (51.7)	
(25–50%)	161 (20.5)	156 (21.5)	5 (8.6)	
(>50%)	28 (3.6)	28 (3.8)	0 (0)	
Capillary tuft necrosis, *n* (%)				0.137
None	704 (89.8)	653 (89.9)	51 (87.9)	
(<25%)	71 (9.1)	66 (9.1)	5 (8.6)	
(25–50%)	8 (1.0)	7 (1.0)	1 (1.7)	
(>50%)	1 (0.1)	0 (0)	1 (1.7)	
Subendothelial hyaline deposits, *n* (%)				
None	333 (44.7)	307 (44.5)	26 (47.3)	0.559
Segmental	194 (26.0)	183 (26.5)	11 (20.0)	
Diffuse	218 (29.3)	200 (29.0)	18 (32.7)	
Interstitial leukocyte infiltration, *n* (%)				0.052
None	196 (25.0)	189 (26.0)	7 (12.3)	
<25%	461 (58.9)	427 (58.8)	34 (59.6)	
25–50%	87 (11.1)	76 (10.5)	11 (19.3)	
50–75%	30 (3.8)	26 (3.6)	4 (7.0)	
>75%	9 (1.1)	8 (1.1)	1 (1.8)	
Interstitial fibrosis, *n* (%)				0.023
None	330 (42.1)	315 (43.4)	15 (26.3)	
<25%	367 (46.9)	336 (46.3)	31 (54.4)	
25–50%	63 (8.0)	56 (7.7)	7 (12.3)	
50–75%	17 (2.2)	13 (1.8)	4 (7.0)	
>75%	6 (0.8)	6 (0.8)	0 (0)	
Tubular necrosis, *n* (%)	43 (5.5)	38 (5.2)	5 (8.8)	0.260
Tubular atrophy, *n* (%)				0.035
None	326 (41.6)	313 (43.1)	13 (22.8)	
<25%	360 (46.0)	326 (44.9)	34 (59.6)	
25–50%	73 (9.3)	65 (9.0)	8 (14.0)	
50–75%	19 (2.6)	19 (2.6)	2 (3.5)	
>75%	3 (0.4)	3 (0.4)	0 (0)	
Artery wall thickening, *n* (%)	231 (29.5)	203 (28.0)	28 (48.3)	0.001

### Patient Overall Survival and Kidney Survival

Flow chart of the enrollment, follow-up process, and study outcome is shown in Figure 1. At the end of the study period, 114 patients died; 27 belonged to the late-onset LN group and 87 in the early-onset LN group. The main causes of death were CVD (late vs. early: 22.2% vs. 19.8%, *p* = 0.12) and infection (late vs. early: 22.2% vs. 25.6%, *p* = 0.28). More patients with late-onset LN died of kidney failure (after the onset of ESRD) (late vs. early: 14.8% vs. 4.7%, *p* = 0.02).

**Figure 2:**
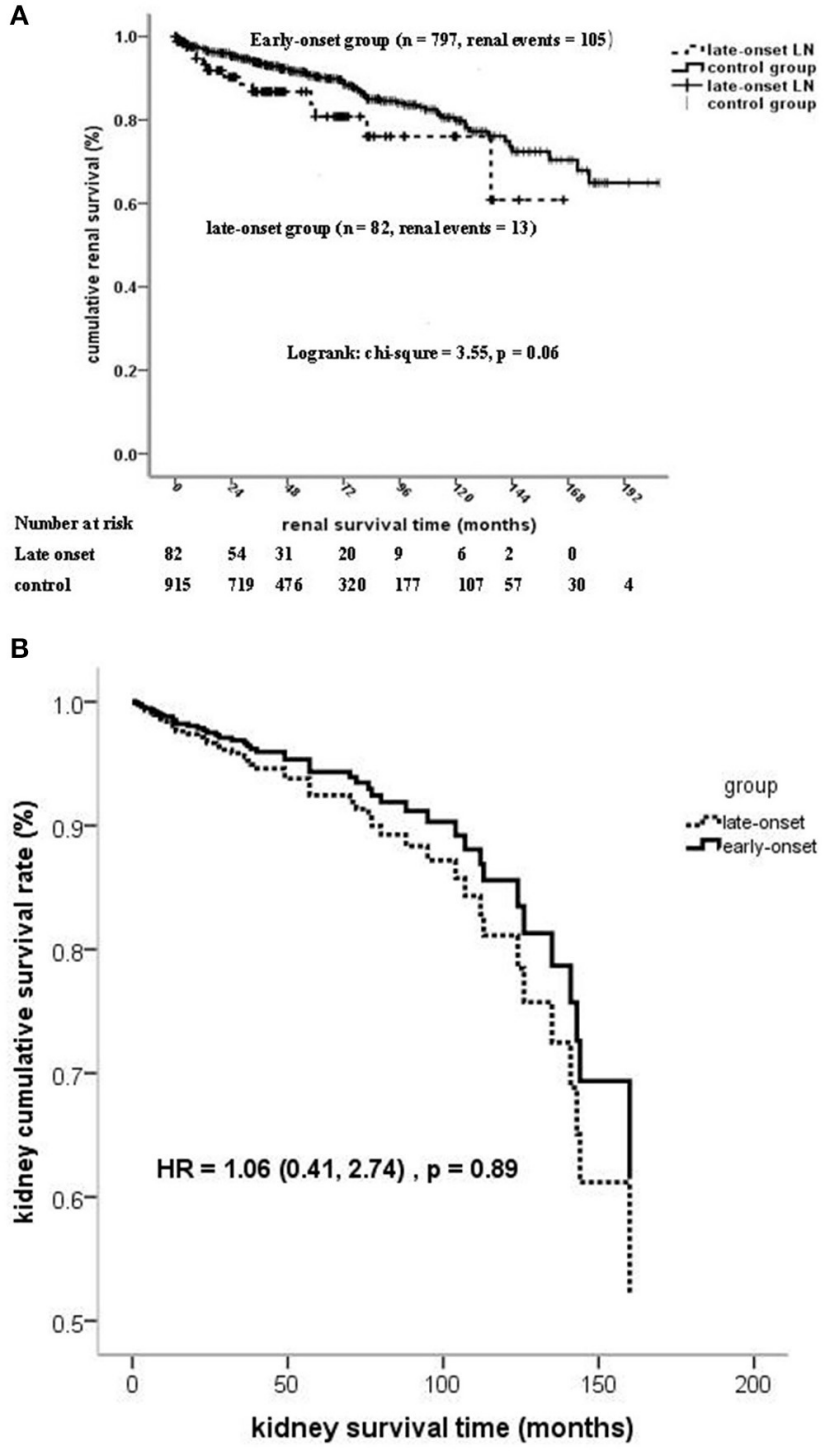
**(A)** Kidney survival (at the composite endpoint of serum creatinine doubling and ESRD) stratified on the age of LN onset. **(B)** Kidney survival (at the composite endpoint of serum creatinine doubling and ESRD) stratified on the age of LN onset by cox regression.

Kaplan–Meier analysis showed that the patient survival of the late-onset LN group at 5 and 10 years (67.6 and 50.5%, respectively) was much poorer than that of the early-onset LN group (89.8 and 84.6%, respectively) (log-rank chi-square = 35.9, *p* < 0.001; Supplementary Figure 2A). However, no significant statistical differences were observed on kidney survival at the composite endpoint of serum creatinine doubling or ESRD (log-rank chi-square = 3.55, *p* = 0.06; Figure 2).

### Association of the Onset Age of LN With Kidney Prognosis

The onset age of LN ≧50 years was an independent risk factor of mortality after adjusted for confounders, such as gender, life expectancy, comorbidity diseases, pathological characteristics, and treatment. No statistically significant association was found between the onset age of LN and kidney failure by using regression models 1 to 4 (Table 5 and Figure 2B).

**Table 5:** Association of late-onset LN with all-cause mortality and kidney mortality.

**Model**	**All-cause mortality**		**Kidney outcome**	
	**HR [CI (95%)]**	***p***	**HR [CI (95%)]**	***p***
Univariable Cox model	1.72 (1.32, 2.24)	<0.001	1.66 (0.93, 2.97)	0.08
Model 1	2.27 (2.10, 5.08)	<0.001	1.25 (0.69, 2.25)	0.45
Model 2	2.16 (1.18, 3.94)	0.012	1.17 (0.53, 2.57)	0.68
Model 3	2.63 (1.35, 5.13)	<0.001	1.11 (0.50, 2.46)	0.78
Model 4	3.03 (1.39, 6.58)	0.005	1.06 (0.41, 2.74)	0.89

*Reference, LN onset age <50; HR, hazard ratio; CI, confidence interval*.

*Model 1: adjusted for sex, life expectancy*.

*Model 2: adjusted for Model 1 and creatinine, uric acid, urine protein, C3, SLEDAI score*.

*Model 3: adjusted for Model 2 steroid dose and immunosuppression drugs*.

*Model 4: adjusted for Model 3 and crescent, glomerular sclerosis, interstitial fibrosis, tubular atrophy*.

### Risk Factors for Kidney Survival in Patients With Late-Onset LN

Further analysis was performed focusing on the risk factors for kidney survival in patients with late-onset LN. The results showed that the increased serum creatinine at baseline was an independent risk factor (hazard ratio = 1.45 (1.20–1.73), *p* < 0.001; Table 6).

**Table 6:** Predictors of kidney survival in patients with late-onset LN.

**Variants**	**Univariate**	***p***	**Multivariate**	***p***
	**HR [CI (95%)]**		**HR [CI (95%)]**	
Gender (F: refer)	2.26 (0.28, 18.09)	0.442		
CVD history	2.79 (0.57, 13.55)	0.202		
Diagnose duration (↑12 months)	0.97 (0.93, 1.02)	0.341		
Urine protein (↑1 g/L)	1.14 (0.94, 1.37)	0.168		
SBP (↑10 mmHg)	1.01 (0.98, 1.04)	0.472		
Oliguria/anuria (no: refer)	3.42 (0.96, 12.18)	0.057	-	NS
Hemoglobin (↑10 g/L)	0.68 (0.51, 0.91)	0.011	-	NS
Microscopic hematuria (no: refer)	1.53 (0.43, 5.47)	0.506		
Baseline creatinine (↑100 μmol/L)	1.38 (1.13, 1.69)	0.001	1.45 (1.20, 1.73)	<0.001
Baseline uric acid (↑100 mmol/L)	1.00 (1.00, 1.01)	0.059	–	NS
Serum albumin (↓10 g/L)	0.93 (0.84, 1.04)	0.099	–	NS
AKI (no: refer)	2.13 (0.62, 7.32)	0.229		
C3 (↓0.1 g/L)	0.66 (0.05, 8.76)	0.759		
hs-CRP (↑1 g/L)	1.00 (1.00, 1.01)	0.018	-	NS
SLEDA score (↑1)	1.06 (0.96, 1.18)	0.211	-	NS
Glomerular sclerosis (↑1%)	15.1 (0.55, 412.3)	0.108	-	NS
Crescents (↑1%)	0.62 (0.007, 55.8)	0.837		
Interstitial fibrosis>75% (no: refer)	1.88 (0.21, 16.8)	0.573		
Tubular necrosis (no: refer)	24.1 (0.00, 165.3)	0.575		
Tubular atrophy (no: refer)	1.31 (0.54, 3.14)	0.541		
Artery wall thickening (no: refer)	2.16 (0.42, 11.18)	0.355		
Glucocorticoid at initial treatment	0.30 (0.04, 2.41)	0.259		
Immune suppressor at initial treatment	1.06 (0.29, 3.78)	0.926		

*CVD, Cardiovascular disease; SBP, systolic blood pressure; AKI, acute kidney injury; hs-CRP, high-sensitivity C-reactive protein; SLEDAI, systemic lupus erythematosus disease activity index*.

## Discussion

Late-onset SLE is a relatively minority in the SLE patients. Studies have shown differences on clinical manifestation, seroantibodies and damage accrual between late-onset and the early-onset, but the findings were variable (2–9). Our large retrospective cohort study indicated that the patient survival of late-onset LN population was poorer while the kidney outcome was comparable than that of patients with early-onset LN. The advanced age-onset of LN was closely related to the poor clinical outcome independent of potential confounders.

Late-onset SLE has been reported to occur in 3%−18% of patients with SLE. As early as 1989, a meta-analysis conducted by Ward and Polisson concluded that the clinical manifestation between early- and late-onset SLE is different (2). However, there remains a debate on the clinical characteristics and the outcomes of patients with late-onset SLE (21–25). The discrepancy in findings in previous studies may partially be due to the different cutoff ages used in different studies and the inter-ethnic differences (13, 25). In our cohort, we selected a cut-off age of 50 years because it was arbitrarily designated as late onset when the clinical diagnosis of the disease occurred after the age of 50 years (14, 26, 27). Based on the cut-off age, the incidence of late-onset LN was 8.1% in our cohort. The percentage of male in the late-onset SLE group doubled that in the early-onset SLE group, which may be explained by the less apparent female predominance among older patients with SLE, mainly owing to the decreased estrogen levels (26). Estrogen status is important in determining disease activity and prognosis in SLE (21, 26, 28), and sex and age factors may inevitably interact to influence SLE prognosis. However, when we specifically adjusted for sex as a confounder in the multivariate Cox regression model, the hazard ratio of death in patients with late-onset LN was three-fold higher than that in patients with early-onset LN. This may suggest the age plays an important role on patient survival independent of sex difference.

Previous studies reported that late-onset SLE has greater comorbidities (8, 14), lower SLE activity (14), and less major organ involvement (8, 12). As shown in our study, patients with late-onset LN had higher blood pressure, more CVD complications, and worse kidney function at baseline, while most systematic organ involvement associated with SLE are not different from the early-onset group. Moreover, the SLEDAI scores and the SDI scores were also similar in the two groups, which is different from previous report on a lower SLEDAI score in patients with late-onset SLE (8, 14). Further analysis of kidney histopathology implied that the chronic lesions (glomerular sclerosis, interstitial fibrosis, tubular atrophy, and cortex wall thickening) were more severe in patients with late-onset LN. However, active lesions such as crescents, karyorrhexis, capillary tuft necrosis, glomerular capsule adhesion, tubular necrosis, were not significantly different between the two groups. The glomerular leukocyte infiltration and endocapillary hypercellularity showed more severe in the early-onset group. Therefore, it indicated that the relatively poorer conditions of patients with late-onset SLE at baseline are mainly associated with their advanced age, while not with SLE activity *per se*. To our knowledge, there is only few detailed information available on kidney histopathological features of late-onset LN in published papers (3, 23, 24). Both chronic lesion and active lesion associated influenced the outcome of LN. Therefore, the results of kidney survival analysis which showed no statistical difference even by Cox regression (adjusted for multivariate) was the combined effect of the two factors. In consideration of the more comorbid conditions and chronic impairment of histopathology, practitioners tended to give late-onset LN with lower corticosteroid dose and less frequent cyclophosphamide lest the side-effect. However, the present study failed to prove whether the limited therapeutic options for SLE due to the presence of comorbidities and concomitant therapies in elderly patients may contribute to the poorer outcome.

There were conflicting reports with regard to patient outcome of late-onset SLE. An epidemiological study from Canada (29) and long-term cohort studies from Brazil (7) and China (28) found that late-onset SLE is not a benign subgroup. Another study reported that older patients have less kidney involvement and better prognosis than their younger counterparts (9). In our cohort, the 5- and 10-year survival rates of patients with late-onset LN were 68.5 and 49.1%, respectively, which are much lower than those of patients with early-onset LN (95.5 and 92.1%). In patients with late-onset LN, kidney failure was a more common cause of death (14.8%) than in patients with early-onset LN (4.7%). Results of multivariate regression equation showed that onset at age >50 years was an independent risk factor of patient survival. Considering that age is naturally associated with survival, life expectancy was taken as a confounder into the multivariable Cox regression model. The independent association between late-onset LN and patient survival still existed, which made the conclusion more robust.

Only a small number of studies have reported on kidney outcome (3, 30, 31). In our study, although patients with late-onset LN had a poorer kidney baseline, no significant difference on the kidney outcome was found between late- and early-onset LN. The contradiction of worse kidney baseline and benign kidney prognosis may have partially been related to relatively milder SLE course in older patients (12). These might also be explained by the results of comparisons on histopathological characteristics. In general, no significant difference was found in the pathological type distribution between the two groups. With respect to the activity indices and chronicity indices, there were significantly lower scores in activity indices but significantly higher scores in chronic indices such as tubular atrophy and interstitial fibrosis in the late onset group. This is consistent with another report based on Chinese population (3). Nevertheless, in older patients, AKI occurred, to some extent, more frequently on conditions of aggravating factors, such as hypovolemia, infection, and nephrotoxic medication. More attention should be paid on overall condition balancing and complication correcting in elder patients.

The strengths of our study are the relatively large sample size and long follow-up duration focusing on the kidney survival and taking a closer sight on the detailed pathological changing analysis to investigate the effect of onset-age of LN on the prognosis of late-onset LN.

An important potential limitation of our study is its retrospective design, where information bias, selection bias, and uncontrolled confounding effects could potentially influence the results. A critical and unresolved question is whether or not the poorer outcomes observed in patients with late-onset LN are solely age related. This issue warrants further investigation in a prospective controlled cohort study.

## Conclusions

Age-related impairments are the most significant determinant of both clinical and pathological manifestations in patients with late-onset LN. Patients with late-onset LN have milder active lesions related to SLE but severer chronic lesions in kidney pathology, and they have an unfavorable patient outcome but rather acceptable kidney prognosis.

## Data Availability Statement

The raw data supporting the conclusions of this article will be made available by the authors, without undue reservation.

## Ethics Statement

The study was approved by the Human Ethics Committee of the First Affiliated Hospital, Sun Yat-sen University. Each participant provided written informed consent.

## Author Contributions

WC and XY designed the study. NT drafted the manuscript. NT, PRY, and QML participated in the data collection. WFC reviewed the pathology slices. NT, QZ, and LYH performed the statistical analysis. All authors read and approved the final manuscript.All authors contributed to the article and approved the submitted version.

## Funding

This work was supported by grants from the National Natural Science Foundation of China (Nos. 81970599, 82170737, and 81960144); Key Laboratory of National Health Commission, and Guangdong Provincial Key Laboratory of Nephrology, Guangzhou, China (Nos. 2002B60118 and 2020B1212060028).

## Conflict of Interest

The authors declare that the research was conducted in the absence of any commercial or financial relationships that could be construed as a potential conflict of interest.

## Publisher's Note

All claims expressed in this article are solely those of the authors and do not necessarily represent those of their affiliated organizations, or those of the publisher, the editors and the reviewers. Any product that may be evaluated in this article, or claim that may be made by its manufacturer, is not guaranteed or endorsed by the publisher.
