# Corrigendum: Long-term kidney prognosis and pathological characteristics of late-onset lupus nephritis

**DOI:** 10.3389/fmed.2022.1018311

**Published:** 2022-09-05

**Authors:** Na Tian, Qian Zhou, PeiRan Yin, WenFang Chen, LingYao Hong, QiMei Luo, MengHua Chen, XueQing Yu, Wei Chen

**Affiliations:** ^1^Department of Nephrology, The First Affiliated Hospital, Sun Yat-sen University, Key Laboratory of National Health Commission, Guangdong Provincial Key Laboratory of Nephrology, Guangzhou, China; ^2^Department of Nephrology, General Hospital of Ningxia Medical University, Ningxia, China; ^3^Department of Medical Statistics, Clinical Trials Unit, The First Affiliated Hospital of Sun Yat-sen University, Guangzhou, China; ^4^Department of Nephrology, Second Affiliated Hospital of Soochow University, Suzhou, China; ^5^Department of Pathology, The First Affiliated Hospital, Sun Yat-sen University, Guangzhou, China; ^6^Guangdong Provincial People's Hospital, Guangzhou, China

**Keywords:** lupus nephritis, late-onset, kidney outcome, renal pathology, Chinese population

In the published article, there was an error in affiliation 1. Instead of “Key Laboratory of National Health Commission, Guangdong Provincial Key Laboratory of Nephrology, Guangzhou, China,” it should be “Department of Nephrology, The First Affiliated Hospital, Sun Yat-sen University, Key Laboratory of National Health Commission, Guangdong Provincial Key Laboratory of Nephrology, Guangzhou, China.”

Affiliation 7 has been deleted.

The authors apologize for this error and state that this does not change the scientific conclusions of the article in any way. The original article has been updated.

## Publisher's note

All claims expressed in this article are solely those of the authors and do not necessarily represent those of their affiliated organizations, or those of the publisher, the editors and the reviewers. Any product that may be evaluated in this article, or claim that may be made by its manufacturer, is not guaranteed or endorsed by the publisher.

