# Supplementary material for: Long-Term Kidney Prognosis and Pathological Characteristics of Late-Onset Lupus Nephritis
Source: Front Med (Lausanne). 2022 May 30;9:882692. doi: 10.3389/fmed.2022.882692 (PMC9197116; doi:10.3389/fmed.2022.882692)
Supplement: Supplementary file 1 [file Data_Sheet_1.docx]

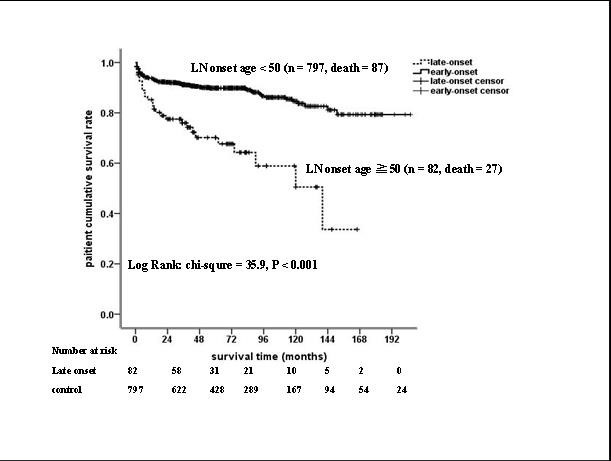


Patient survival stratified on the age of LN onset


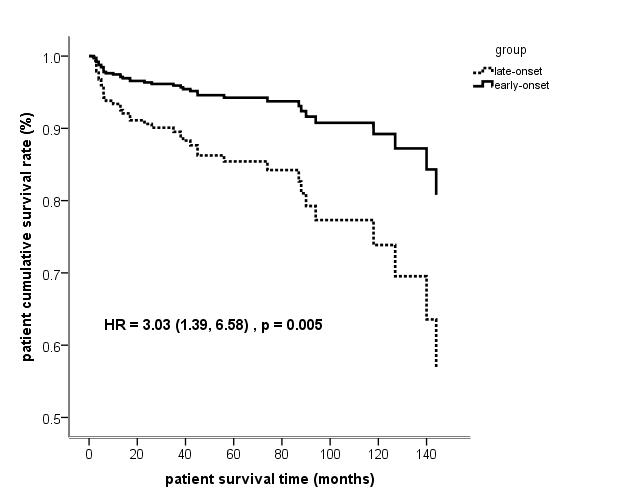


Patient survival stratified on the age of LN onset by cox regression
